# Antifungal properties of a new terpernoid saponin and other compounds from the stem bark of *Polyscias fulva* Hiern (Araliaceae)

**DOI:** 10.1186/s12906-015-0541-7

**Published:** 2015-02-15

**Authors:** Guy Sedar Singor Njateng, Zhizhi Du, Donatien Gatsing, Arno Rusel Nanfack Donfack, Michel Feussi Talla, Hippolyte Kamdem Wabo, Pierre Tane, Raymond Simplice Mouokeu, Xiaodong Luo, Jules-Roger Kuiate

**Affiliations:** Laboratory of Microbiology and Antimicrobial Substances, Faculty of Science, University of Dschang, P.O. Box 67, Dschang, Cameroon; Laboratory of Natural Product Chemistry, Faculty of Science, University of Dschang, P.O. Box 67, Dschang, Cameroon; Institute of Fisheries and Aquatic Sciences, University of Douala, Cameroon, P.O Box 7236, Douala, Cameroon; State Key Laboratory of Phytochemistry and Plant Resources in West China, Kunming Institute of Botany, Chinese Academy of Sciences, Kunming, 650204 People’s Republic of China

**Keywords:** *Polyscias fulva*, Compounds, Antifungal activity, Yeasts, Dermatophytes

## Abstract

**Background:**

In our previous studies, it was evident that the dichloromethane-methanol (1:1 v/v) stem barks extract of *Polyscias fulva* and fractions (ethyl acetate, *n-*butanol and residue) demonstrated interesting antidermatophytic activities. So, as a continuity of that, this work aimed at identifying active principles with antifungal properties from *P. fulva* that could be used as markers for possible standardization of this plant as phytomedicine.

**Methods:**

The ethyl acetate, *n*-butanol and residual fractions of the dichloromethane-methanol (1:1 v/v) stem bark extract of *Polyscias fulva* were further fractionated by column chromatography and the structures of isolated compounds elucidated based on their spectroscopic data in comparison with existing literature information. Antifungal activity was assayed by broth microdilution techniques on yeasts and dermatophytes spores.

**Results:**

The fractionation of the crude dichloromethane-methanol (1:1 v/v) stem bark extract of *Polyscias fulva* led to the isolation of 10 known compounds (**1** to **10**) and one new saponin (**11**: 3-*O*-[*α*-L-rhamnopyranosyl (1–2)-*α*-L-arabinopyranosyl]-28-*O*-[*α*-L-4-*O*-acetyl-rhamnopyranosyl (1–4)-*β*-D-glucopyranosyl-(1–6)-*β*-D-glucopyranosyl]-hederagenin). Among these compounds, 3-*O*-*α*-L- arabinopyranosyl-hederagenin and 3-*O*-[*α*-L-rhamnopyranosyl (1–2)-*α*-L-arabinopyranosyl]-hederagenin were the most active on the tested fungi with MIC values ranging from 0.78 to 100 μg/ml against both yeasts and dermatophytes.

**Conclusion:**

The results of this work constitute a step forward in the possible development of an antidermatophytic phytomedicine from *Polyscias fulva* stem bark, the isolated compounds being possible markers for the standardisation.

**Electronic supplementary material:**

The online version of this article (doi:10.1186/s12906-015-0541-7) contains supplementary material, which is available to authorized users.

## Background

Exploring the healing power of plants is an ancient concept. For many centuries people have been trying to alleviate and treat diseases with different plant extracts and formulations [1]. The interest in plants with antimicrobial properties has been revived because of current problems associated with the use of antibiotics [2]. The fact that microorganisms among which fungi nowadays tend to develop resistance towards drugs, coupled to the undesirable side effects of certain antibiotics is a real problem of concern. Medicinal plants constitute a prolific source of antimicrobial substances. The valorization of medicinal plants through the production of phytomedicine implies the isolation of active compounds that can be used in the standardization process of such drugs.

*Polyscias fulva* is a medium size and fast growing deciduous tree of the tropical forests of sub-Saharan Africa which is found at an altitude range of 1,180-2,500 m, with annual rainfall of 1,500-2,000 mm [3]. In Cameroon, decoction of its bark is orally administered to cure venereal infections [4] while paste from its stems barks and leaves are used topically against dermatoses. In previous studies, the dichloromethane extract from the bark of *Polyscias fulva* appeared to possess a weak antiplasmodial against *Plasmodium falciparum* (IC_50_ = 9.8 μg/ml) and antitrypanosomial activities against *Trypanosoma rhodesiense* (MIC = 100 μg/ml) [5]. Furthermore, its dichloromethane-methanol (1:1 v/v) extract showed interesting *in vitro* and *in vivo* antidermatophytic properties [6]. With the aim of producing a standardized phytomedicine from the plant species, the dichloromethane-methanol of the stem bark was fractionated to isolate and characterize the antifungal active principles.

## Methods

### Materials

#### Plant material

The stem bark of *Polyscias fulva* (Hiern) was collected in April 2008 at Bazou (Nde Division, West Region, Cameroon). Botanical identification was done at the Cameroon National Herbarium in Yaoundé by Mr Tadjouteu Fulbert, where a voucher specimen was kept under the reference number 43546/HNC.

### Microorganisms

The antimicrobial activities of different substances were carried out on seven yeasts and eleven dermatophytes. Yeasts consisted of *Candida albicans* (ATCC 1663)*, C. krusei* (ATCC 6258), *C. parapsilosis (*ATCC 22019)*, C. lucitaniae* (ATCC 200950), *C. glabrata* (IP 35), *Cryptococcus neoformans* (IP 95026) and *Candida guilliermondii* (clinical isolate). Dermatophytes were made up of *Microsporum audouinii*, *Trichophyton rubrum*, *Trichophyton ajelloi* and *Trichophyton equinum* (clinical isolates) and *Trichophyton mentagrophytes* (E 1425), *Trichophyton terrestre* (E 1501), *Microsporum gypseum* (E 1420) and *Epidermophyton floccosum* (E 1423); *T. violaceum* (CBS201.88), *Microsporum canis (*CBS113480) and *M. ferrugeneum (*CBS457.80)*.*

The references strains ATCC were obtained from the American Type Culture Collection (Rockville, MD, USA), IP from “Institut Pasteur” of Paris-France, E from “Ecole Nationale Vétérinaire d’Aford” in France, CBS from the *Centraalbureau Voor schimmelcultures* (Central office for fungal cultures) in Netherlands, whereas the clinical isolate were obtained from the Laboratory of Bacteriology and Mycology of the “Centre Pasteur” of Yaoundé-Cameroon. The strains have been maintained in the refrigerator at 4°C on agar slant.

#### Phytochemical materials

Globally column chromatography (CC) was performed on silica gel (80–120 and 200–300 mesh, Qingdao Marine Chemical Co., China), Hp-20 (40–63 μm, Merck), and Sephadex LH-20 (GE Healthcare, Sweden). TLC was performed on HSGF_254_ (0.2 mm, Qingdao Marine Chemical Co., China) or Rp-18 F_254_ (0.25 mm, Merck). Fractions were monitored by TLC and spots were visualized by heating silica gel plates sprayed with 10% H_2_SO_4_ in EtOH. Semipreparative HPLC was run on Agilent 1100 liquid chromatograph with diode array detector (DAD), Zorbax-SB-C18 column (5 μm; 25 cm × 9.4 mm i.d.).

UV spectra were measured using a Shimadzu UV-2401 PC spectraphotometer. IR spectra were obtained on Bruker Tensor-27 infrared spectrophotometer with KBr pellets. ESI-MS spectra were recorded on a Bruker HTC/Esquire spectrometer, HRESIMS spectra were recorded on an API Qstar Pulsar instrument. NMR, ^1^H-^1^H COSY, HMBC, and HSQC experiments were performed on Bruker AM-400, DRX-500, and Avance III 600 instruments with TMS as the internal standard. Chemical shifts (*δ*) were expressed in ppm with reference to the solvent signals.

### Methods

#### Fractionation and isolation of active compounds from the plant extract

The dichloromethane-methanol (1:1 v/v) extract (263 g) from the stem bark of *Polyscias fulva* was pre-dissolved in 250 ml of methanol and water (1:9) and shaken vigorously in 500 ml of *n*-hexane. The *n*-hexane phase was collected and the process repeated twice. The methanol was then evaporated from the polar phase. The residue obtained after methanol evaporation was partitioned in the ethyl acetate and finally in the *n*-butanol as above. The n-hexane, ethyl acetate and *n*-butanol phases were concentrated under reduced pressure in a rotatory evaporator to obtain 24.25 g of the *n*-hexane (09.22%), 23.77 g of the ethyl acetate (09.03), 16 g of the *n*-butanol (06.08%) and 195.98 g of the residual (74.52%) fractions after solvent evaporation. The ethyl acetate, *n*-butanol and residual fractions were fractionated by column chromatography.

#### Column fractionation of ethyl acetate fraction

20.77 g of ethyl acetate fraction was subjected to a silica gel column (60×8.5 cm) chromatography eluted gradiently with CHCl_3_–MeOH to give eight fractions [F1 (4.5 g), F2 (2 g), F3 (2.4 g), F4 (2.7 g), F5 (2.0 g), F6 (0.6 g), F7 (4.0 g), F8 (0.3 g)]. Antimicrobial activity was concentrated in fractions F1 and F7 eluted with CH-Cl_3_ and CH-Cl_3_-MeOH (1:1) respectively. Further silica gel column purification of F1 (42×3 cm) eluted with petroleum ether-CHCl_3_ in a gradient elution mode and F7 (38x3 cm) eluted with CHCl_3_-MeOH in a gradient elution mode yielded five sub-fractions each denoted F1.1 to F1.5 and F7.1 to F7.5 respectively. F1.1 (135 mg), F1.5 (103 mg), F7.5 (2.62 g) were the most active against the tested microorganisms and were then subjected to further purification. Column purification of F1.1 (120 mg) on Sephadex gel LH-20 (140×2.5 cm) with CHCl_3_-MeOH (1–1) as eluting system afforded compound **2** (50 mg). Purification of F1.5 (90 mg) was done through a Sephadex column LH-20 (146x2.5 cm) and afforded compound **1** (16 mg). Fraction F7.5 (2.39 g) was further applied to chromatographic silica gel column (25×2cm) and eluted with CHCl3-MeOH (25:75) to obtain 5 sub-fractions (F7.5.1 to F7.5.5). F7.5.1, F7.5.2, F7.5.5, having antimicrobial activity, were further purified. F7.5.1 (457 mg) was purified on silica gel column (30×1.5 cm) with CHCl3-MeOH (30:70) as eluting solvent. This purification step afforded compound **3** (20 mg). Sub-fraction F7.5.2 (378 mg) was subjected to preparative TLC plate with CHCl_3_-MeOH (20:80) as eluting system and then to Sephadex LH-20 column (120×1.5 cm) with CHCl_3_-MeOH (10:90) as eluting system to afford compound **4** (50 mg). The sub-fraction F7.5.5 (504 mg) was applied to chromatographic silica gel (80–120 μm) columns (20x1.5 cm) and eluted with CHCl_3_-MeOH-H_2_O (30:69:1) to obtain compounds **5** (29 mg) and **6** (19 mg).

#### Column fractionation of *n*-butanol fraction

The *n*-butanol fraction (13 g) of the crude dichloromethane-methanol (1:1 v/v) extract of *P. fulva* stem bark was fractionated using a chromatographic column HP 20 (60x8.5 cm) with MeOH-H_2_O (1:0 → 0:1 v/v) followed by acetone. 25 fractions of 250 ml each were collected and pooled into 4 major fractions (N1 to N4) after evaporation in vacuum. Fractions N3 (7.10 g) and N4 (3.09 g) presented antimicrobial activity and then were subjected to further purifications.

N3 was subjected to silica gel column (74x4 cm) chromatography and eluted with EtOAc-MeOH (1:0 → 0:1 v/v). Based on the TLC profiles in EtOAc-MeOH, this afforded 7 sub-fractions (N3.1 to N3.7) of which N3.5 (4.09 g) and N3.7 (2.1 g) were active against the microorganisms. N3.5 was then fractionated using Sephadex LH-20 column (103×3 cm) with MeOH as eluting solvent. A total of 142 fractions of 10 ml each were collected and grouped into 4 sub-fractions (N3.5.1 to N3.5.4) based on their thin layer chromatograms. N3.5.2 (2.64 g) was the most active and was applied to silica gel (80–120 μm) column (19×2 cm). It was eluted with EtOAc-MeOH (1:0 → 0:1 v/v) and afforded 7 sub-fractions (N3.5.2.1 to N3.5.2.7). Sub-fractions N3.5.2.1 (0.61 g), N3.5.2.2 (0.62 g) and N3.5.2.6 (0.74 g) were biologically active. N3.5.2.1 was applied to silica gel column (35×2 cm) and eluted in an isocratic elution mode using EtOAc-MeOH (18:1). From this, 4 sub-fractions (N3.5.2.1.1 to N3.5.2.1.4) were obtained. Sub-fractions N3.5.2.1.1 and N3.5.2.1.3 were subjected to gel permeation on Sephadex LH-20, and eluted with MeOH to yield compounds **7** (40 mg) and **8** (52 mg) respectively. N3.5.2.2 was applied to silica gel column (40×2.5 cm) and eluted in an isocratic elution mode using EtOAc-MeOH-H_2_O (18:5:1) to give 6 sub-fractions (N3.5.2.2.1 to N3.5.2.2.6) based on their TLC profiles. Subjected to Sephadex LH-20 column chromatography, N3.5.2.2.2 gave compound **10** (103 mg). N3.5.2.6 (456 mg) was also applied to silica gel column (45×2 cm), eluted with EtOAc-MeOH-H_2_O 18:6:1, and gave 3 sub-fractions (N3.5.2.6.1 to N3.5.2.6.3). N3.5.2.6.3 (236 mg) was further purified by semi-preparative HPLC (MeOH-H_2_O, 25:75 v/v) to afford compound **11** (130 mg, Rt = 27.7 min). Sub-fraction N3.7 was separated by MPLC or Medium pressure liquid chromatography (Büchi pump manager C-615) under the following conditions: pressure = 4.6 bar, flow rate = 20 ml/min, time = 20 min, volume collected = 500 ml. This was eluted with H_2_O-MeOH (1:0 → 0:1 v/v) to obtain 5 sub-fractions (N3.7.1 to N3.7.5). Among these sub-fractions, N3.7.5 (509 mg) was bioactive and was applied to a silica gel column chromatography (CHCl_3_–MeOH, 80:20 v/v) to give 89 sub-fractions of 10 ml each grouped into 3 major sub-fractions (N3.7.5.1 to N3.7.5.3). N3.7.5.3 (32 mg) was further subjected to Sephadex LH-20 (103×1.5 cm) with MeOH as eluting system to give compound **9** (12 mg).

N4 (3.09 g) was applied to silica gel column (45x3 cm), eluted with EtOAc-MeOH (1:0 → 0:1 v/v) to obtain 96 fractions of 300 ml each gathered into 14 sub-fractions (N4.1 to N4.14). The most bioactive sub-fraction (N4.1, 750 mg), was applied to silica gel column (50×2 cm) with EtOAc-MeOH (6:1) as eluent to give 4 sub-fractions (N4.1.1 to N4.1.4). N4.1.4 (401 mg) was separated by preparative silica gel plate using CHCl_3_-MeOH-H_2_O (60:9:1 v/v/v) as eluting system to have 2 sub-fractions (N4.1.4.1 and N4.1.4.2). 4.1.4.2 (207 mg) was applied to silica gel column (30×2 cm) and eluted with CHCl_3_–MeOH-H_2_O (240:9:1 v/v/v). 169 fractions were collected, and grouped into 4 sub-fractions (N4.1.4.2.1 to N4.1.4.2.4). Compounds **5** (11 mg) and **6** (13 mg) crystallized from sub-fractions N4.1.4.2.2 and N4.1.4.2.4 respectively.

#### Column fractionation of residual fraction

The residual fraction (190.98 g) of the crude extract of *P. fulva* stem bark was fractionated on a chromatographic column HP 20 (70×8.5 cm) using MeOH-H_2_O (1:0 → 0:1 v/v) as eluting system followed by acetone. 25 fractions of 250 ml each were collected and pooled on the basis of their TLC profiles (in EtOAc-MeOH-H_2_O) into 6 major fractions (R1 to R6). After evaporation in vacuum, R6 (9.50 g) which was more active was subjected to further purifications. Sub-fraction R6 was fractionated on a chromatographic column HP 20 (50×8.5 cm). Elution started with H_2_O followed by gradual increases of MeOH to afford 92 individual fractions (250 ml each) which were grouped into eight collective fractions (R6.1 to R6.8). Fraction R6.8 (1.03 g), which was the most bioactive, was subjected to separation over silica gel (Sigma, 80–120 μm) column (50×2.50 cm) using a gradient of CHCl_3_-MeOH-H_2_O to give 6 main sub-fractions (R6.8.1 to R6.8.6). Sub-fraction R6.8.1 (509 mg), the most bioactive, was purified by silica gel column (50×2 cm), eluted with the isocratic solvent system EtOAc-MeOH-H_2_O (18:5:1) to give compound **10** (75 mg) from sub-fraction R6.8.1.2, while sub-fraction R6.8.1.4 (76 mg) was chromatographed on Sephadex LH-20 and eluted with MeOH to afford compound **11** (40 mg).

Compound **1** is a white crystalline solid (Petroleum ether); ESIMS: *m /z* 219 [M + Na]^+^, 197 [M + H]^+^; Molecular formula: C_10_H_12_O_4_;

^13^C -NMR (100 MHz,CDCl_3_); *δ* (ppm): 172.6 (1-C = O); 163.1 (C- 2); 158.0 (C-4); 140.1 (C-6); 110.5 (C-5); 108.4 (C-1); 105.2 (C-3); 51.8 (MeO-C = O ); 24.1 (Me-C_6_); 7.6 (Me-C_3_). NMR ^1^H (400 MHz, CDCl_3_); *δ* (ppm): 12.05 (s, 1H,); 6.21 (s, 1H, H-5); 3.92 (s, 3H, OCH_3_); 2.45 (s, 3H, −CH_3_); 2.10 (s, 3H, −CH_3_).

Compound **2** is in form of white needles (Petroleum ether); m.p: 137–138°C; Molecular formula : C_29_H_50_O ;

^1^H-NMR (400 MHz,CDCl_3_), *δ* (ppm): 7.26 (s,OH-4); 5.35 (m, 1H, H-6); 3.53 (m,1H, H-3); 1.24 (s, 3H, H-19); 1.17 (s, 3H, H-18); 1.07 (s, 3H, H-26);1.01 (s, 3H, H-27); 0.92 (s, 3H, H-21); 0.90 (s, 3H, H-29).

^13^C-NMR (100 MHz, CDCl_3_) *δ* (ppm):140.7 (C-5); 121.7 (C-6); 71.8 (C-3); 56.7 (C-4); 50.1 (C-17); 45.8 (C-14); 42.3 (C-13); 39.7 (C-10); 33.9 (C-20); 31.9 (C-25); 31.8 (C-26); 28.2 (C-23); 24.3 (C-12); 21.0 (C-18); 19.0 (C-21); 11.9 (C-27).

Compound **3** is a white powder (Cloroform); m.p: 119–120°C; ESIMS : *m/z*: 381 [M + Na]^+^; Molecular formula : C_20_H_22_O_6_ ;

^1^H NMR (600 MHz, C_5_D_5_N); δ (ppm): 11.25 (s, 1H, −OH) ; 7.18 (m, 2H, H-6, 5); 7.16 (d, 1H, H-2); 4.95 (d, 1H, *J* = Hz); 4.35 (d, 1H, *J* = Hz ); 4.05 (d, 1H, *J* = Hz ); 3.88 (3H, s, −OCH_3_); ^13^C-NMR (150 MHz, C_5_D_5_N) (ppm): 149.3 (C-3); 133.6 (C-1); 120.2 (C-6); 116.9 (C-5); 111.3 (C-2); 86.9 (C-7); 72.4 (C-7’); 56.4 (C-8/8’); 55.3 (CH_3_O-C_3_).

Compound **4** is a white powder (Ethyl acetate); m.p.: 171–172°C; ESIMS: *m/z* 479 [M + Na]^+^; Molecular formula: C_30_H_48_O_3_;

^13^C-NMR (150 MHz, CD_3_OD) *δ* (ppm): 182.1 (C-28); 145.4 (C-13); 123.8 (C-12); 79.7 (C-3); 56.9 (C-5); 48.7 (C-9); 47.8 (C-19); 47.4 (C-17); 43.0 (C-17); 42.9 (C-18); 40.7 (C-8); 40.0 (C-4); 39.9 (C-1); 38.3 (C-10); 35.0 (C-21); 34.1 (C-29); 34.0 (C-22); 33.7 (C-7); 31.8 (C-20); 31.0 (C-23); 29.0 (C-23); 28.9(C-15); 28.0 (C-2); 26.5 (C-27); 24.7 (C-11); 24.2 (C-16); 24.1 (C-30); 19.6 (C-6); 17.9 (C-24); 16.5 (C-26); 16.0 (C-25).

^1^H-NMR (600 MHz, CD_3_OD); *δ* (ppm): 5.24 (brs, 1H, H-12); 3.19 (m, 1H, H-18); 2.83 (dd,7.2, 1H, H-3).

Compound **5** is a white amorphous powder (Ethyl acetate); ESI MS: *m/z* 757 [M + Na]^+^; Molecular formula: C_41_H_66_O_11_;

^13^C-NMR (150 MHz, CD_3_OD): 182.1(C-28); 145.2 (C-13); 123.8 (C-12); 105.1 (C-1’); 102.2 (C-1”); 90.7 (C-3); 77.0 (C-2); 75.4 (C-16); 74.0 (C-4”); 73.4 (C-3’); 72.3 (C-3”); 70.4 (C-5”); 68.7 (C-4’); 64.1 (C-5’); 57.3 (C-5); 48.7 (C-9); 47.8 (C-19); 47.4 (C-17); 43.0 (C-17); 42.9 (C-18); 40.7 (C-8); 40.0 (C-4); 39.9 (C-1); 38.3 (C-10); 35.0 (C-21); 34.1 (C-29); 34.0 (C-22); 33.7 (C-7); 31.8 (C-20); 31.0 (C-23); 29.0 (C-23); 28.9(C-15); 28.0 (C-2); 26.5 (C-27); 24.7 (C-11); 24.2 (C-16); 24.1 (C-30); 19.6 (C-6); 17.9 (C-24); 16.5 (C-26); 16.0 (C-25).

Compound **6** is a white amorphous powder (Ethyl acetate); ESIMS: *m/z* 773 [M + Na]^+^; Molecular formula: C_41_H_66_O_12_;

^13^C-NMR (150 MHz, CD_3_OD): 145.2 (C-13); 123.6 (C-12); 105.1(C-1’); 102.2 (C-1”); 90.7 (C-3); 76.9 (C-2); 75.4 (C-16); 74.0 (C-4”); 73.4 (C-3’); 72.3 (C-3”); 72.3 (C-2”); 70.3 (C-5”); 68.7 (C-4’); 64.1 (C-5’); 57.3 (C-5); 48.7 (C-17); 48.3 (C-19); 47.8 (C-9); 42.8 (C-14); 42.2 (C-18); 40.8 (C-8); 40.4 (C-4); 40.1 (C-1); 38.1 (C-10); 36.7 (C-15); 36.3 (C-21); 34.4 (C-29); 33.6 (C-7); 33.0 (C-22); 28.7 (C-23); 27.4 (C-27); 27.2 (C-2); 25.0 (C-30); 24.6 (C-11); 17.9 (C-26); 17.2 (C-24); 16.3 (C-25).

Compound **7** is a white amorphous powder (Ethyl acetate); ESIMS: *m/z* 479 [M + Na]^+^ ,441[M-CH_3_]^+^ ; Molecular formula : C_35_H_56_O_8 ;_

^13^C NMR (100 MHz, MeOD); *δ* (ppm): 181.9 (C-26); 145.2 (C-13); 123.6 (C-12); 106.4 (C-1’); 83.3 (C-3); 74.5 (C-2’); 72.9 (C-3’); 69.8 (C-4’); 66.9 (C-5’); 64.8 (C-23); 49.6 (C-9); 48.6 (C-9); 48.4 (C-19); 48.1 (C-17); 47.6 (C-5); 43.9 (C-4); 43.0 (C-14); 42.7 (C-18); 40.5 (C-1); 37.7 (C-10); 33.6 (C-22); 31.6 (C-20); 26.5 (C-27); 24.0 (C-16); 17.8 (C-26); 16.4 (C-25); 13.4 (C-24).

Compound **8** is a white amorphous powder (ethyl acetate); °C; ESIMS: m/z 750 [M + Na]^+^; Molecular formula: C_41_H_66_O_12_;

^13^C-NMR (125 MHz,C_5_D_5_N), *δ* (ppm): 181.8 (C-28), 145.3 (C-13), 123.0 (C-12), 104.4 (C-1’), 101.9 (C-1”), 82.1 (C-3), 76.6 (C-5’), 73.9 (C-4‘), 73.7 (C-4”), 72.2 (C-5”), 72.0 (C-3’), 70.2 (C-2’), 70.0 (C-4”’), 64.9 (C-23), 48.6 (C-9), 48.4 (C-17), 47.6 (C-19), 43.9 (C-4), 43.9 (C-14), 42.9 (C-18), 40.4 (C-1), 37.6 (C-10), 33.6 (C-22), 31.6 (C-20), 26.5 (C-27), 29.9 (C-16), 23.9 (C-11), 18.0 (C-6/6”), 17.8 (C-26), 16.4 (C-25), 13.9 (C-24).

^1^H NMR (500 MHz, C_5_D_5_N), *δ* (ppm): 5.23 (brs, H-12); 5.10 (s, H-1’); 4.57 (dd, 9.0, 7.4, H-2’); 3.77 (m, H-3”); 3.74 (H-5”); 3.70 (m, H-4”); 4.52 (m, H-2”); 3.44 (m, H-3’); 3.33 (brm, H-5’b); 3.51 (dd, 13.5, 3.5, H-18); 1.65 (sl H-1”); 1.10 (s, H-25); 0.95 (s, H-26); 0.81 (s, H-29); 0.75 (s, H-30).

Compound **9** is a white amorphous powder (Ethyl acetate); C; ESIMS: m/z 831 [M + Na]^+^; Molecular Formula: C_43_H_68_O_14_;

^13^C-NMR (125 MHz,C_5_D_5_N), *δ* (ppm): 177.0 (C-28), 171.4 (C-6’), 144.6 (C-13), 123.9 (C-12), 107.8 (C-1’), 96.2 (C-1”), 89.6 (C-3), 79.9 (C-3’), 79.4 (C-5’), 78.4 (C-3”), 77.7 (C-5”), 75.9 (C-2”), 74.6 (C-2’), 73.2 (C-4’),71.5 (C-4”), 64.9 (C-23), 62.6 (C-6”), 52.6 (CH_3_O-C_6_’) 48.4 (C-9), 47.4 (C-17), 46.6 (C-19), 42.6 (C-4), 42.2 (C-14), 40.3 (C-18), 40.0 (C-1), 37.4 (C-10), 33.5 (C-22 ), 31.3 (C-20 ), 28.7 (C-16), 26.6 (C-27), 23.8 (C-11), 18.9 (C-6); 17.9 (C-24); 17.4 (C-26); 16.0 (C-25).

^1^H NMR (500 MHz, C_5_D_5_N), *δ* (ppm): 6.36 (d, 10.4, H-1”, 4.99 (d, 7.8, H-1”); 4.73 (dd, 9.0, 7.4, H-2’); 4.62 (m, H-4’); 4.57 (m, H-5”); 4.55 (m, H-4”); 4.42 (m, H-2”); 4.30 (m, H-3’); 4.27 (brm, H-5’b); 3.74 (dd, 13.5, 3.5, H-18); 1.61 a (H-1”); 1.10 (s, H-25); 0.95 (s, H-26); 0.92 (s, H-29); 0.86 (s, H-30).

Compound **10** is a white hite amorphous powder (Ethyl acetate); ESIMS: *m/z* 1244 [(M + H) + Na]^+^; Formula: C_59_H_96_O_26_;

^13^C-NMR (125 MHz,C_5_D_5_N), *δ* (ppm): 176.6 (C-28), 144.2 (C-13), 123.0 (C-12), 104.8 (C-1””), 104.2 (C-1’), 102.8 (C-1”), 101.7 (C-1””’), 95.7 (C-1”’), 78.7 (C-3), 77.2 (C-5”’), 76.6 (C-3”’), 76.0 (C-5’), 75.3 (C-2””), 74.2 (C-2”’), 74.0(C-4‘), 73.9 (C-4”), 72.8 (C-5”), 72.6 (C-3’), 72.3(C-2’), 70.0 (C-4”’), 69.8 (C-6”’), 65.1 (C-5’), 61.1 (C-23/6”’), 48.2 (C-9), 47.8 (C-17), 47.1 (C-19), 43.5 (C-4), 43.5 (C-14), 42.2 (C-18), 40.0 (C-1), 37.0 (C-10), 33.1 (C-22), 30.8 (C-20), 26.1 (C-27), 23.9 (C-16), 23.8 (C-11), 18.5 (C-6/6”), 17.6 (C-26), 16.2 (C-25), 13.9 (C-24).

^1^H NMR (500 MHz, C_5_D_5_N), *δ* (ppm): 6.56 (d, 10.4, H-1”); 6.30 (brs, H-1””’); 6.25 (d, 8.1, H-1”’); 5.38 (brs, H-12); 5.10 (s, H-1’); 4.99 ( d, 7.8, H-1””); 4.98^a^ (H-6a””); 4.73 (dd, 9.0, 7.4, H-2’); 4.70 (m, H-4”’/H-6”’); 4.66^a^ ( H-3”/H-2””’); 4.65^a^ (H-3””’); 4.62^a^ (H-4’); 4.59^a^ (H-4””); 4.57^a^ (H-5”); 4.55^a^ (H-4”); 4.42^a^ (H-2”); 4.38^a^ (H-5””’); 4.34^a^ (H-6””); 4.31^a^ (H-4””’); 4.30^a^ (H-3’); 4.27 (brm, H-5’b); 4.17 (m, H-3””/H-5””); 4.10 (m, H-2””); 3.98 (m, H-5”’); 3.95 (m, H-2”’); 3.74 (m, H-3”’); 3.51 (dd, 13.5, 3.5, H-18); 1.70 (d, 6.4, H-6””’); 1.65 a (H-1”); 1.10 (s, H-25); 0.95 (s, H-26); 0.93 (s, H-29); 0.85 (s, H-30).

^a^Unresolved proton resonances.

Compound **11** is a white powder from AcOEt fraction; m.p. 213–215°C; [α]_D_ -38.9 (c 0.1, MeOH); IR (KBr): υ_max_ = 3428, 1731, 1642 cm^−1^; HRESI-MS: 1285 [M + Na]^+^ (Calcd. for C_61_H_98_O_27_Na, 1285.6193); m/z = 1262; ^1^H and ^13^C NMR data are presented in Table 1.

**Table 1:** ^**1**^ **H NMR and** ^**13**^ **C NMR data of compound 11 (400/100 MHz Pyridine-** ***d***_***5***_ **),** ***δ*** **in ppm,** ***J*** **in Hz**

**Position**	**δ**_**C**_	**δ**_**H**_	**(** ***J*** **in Hz)**	**Position**	**δ**_**C**_	**δ**_**H**_	**(** ***J*** **in Hz)**
1	39.9			3-ara-1’	104.5	5.10	(1H, d, *J* = 6.2)
2	26.2	1.15	(2H, s)	2’	76.9	4.43	(
3	81.1	4.24	(1H, d, *J* = 7.6)	3’	73.9	4.10	
4	43.5			4’	69.5	4.29	
5	47.7			5’	65.8	3.68	(1H,s)
						4.24	(1H, d, *J* = 4.0)
6	18.2			Rha-1”	102.2	6.21	(1H, s)
7	33.0	1.27	(1H, s)	2”	72.4	4.63	(1H, d, *J* = 3.3)
8	39.2			3”	72.6		
9	48.2			4”	74.1	4.27	(1H, s)
10	36.8			5”	69.8	4.65	(2H, s)
11	23.8			6”	18.6	1.62	(3H, d, *J* = 4.7)
12	123.0	5.38	(1H, brs)	28-glu-1”’	95.7	6.21	(1H, dl, *J* = 4.2)
13	144.2			2”’	73.9		
14	41.9			3”’	79.9		
15	21.8	2.25	(1H, s)	4”’	70.8		
		1.04	(1H, s)				
16	23.5		(2H, s)	5”’	78.2	4.19	(1H, m)
17	46.1			6”’	69.1	4.16	(1H, d, *J* = 9.1)
						4.65	(1H, s)
18	42.2	3.14	(1H, d, *J* = 10.6)	Glu-1””	104.9	5.00	(1H, d, *J* = 7.8)
19	47.1			2””	75.8		
20	30.8			3””	76.6		
21	33.9	1.06		4””	78.7		
22	33.1	1.83		5””	76.8	3.62	(1H, d, *J* = 9.4)
		1.55					
23	63.9	3.72	(1H,d, *J* = 7.9)	6””	61.2	4.05	(1H, d, *J* = 3.4)
		4.10	(3H, s)			4.18	(1H,d, *J* = 9.1)
24	14.1	1.03	(3H, s)	Rha-1””’	101.7	5.85	(1H, s)
25	16.3	0.94	(3H, s)	2””’	72.5	4.71	(1H,brs)
26	17.6	1.09	(3H	3””’	70.3	4.56	(1H, d, *J* = 2.2)
27	26.3	2.17	(1H, s)	4””’	75.5	3.90	(1H, t, *J* = 8.4)
						4.28	(1H, brs)
28	176.8			5””’	67.5	5.02	(1H, s)
29	33.3	0.83	(3H, s)	6””’	18.2	1.4	(3H, d, *J* = 4.7)
30	23.8	0.85	(3H, s)	-C = O	170.9		
				-CH_3_	21.3	2.01	(3H, s)

### Acid hydrolysis and GC analysis of compound 11

Two milligrams of compound **11** were refluxed with 2 M HCl (1, 4 dioxane/H2O 1:1, 2 ml) on water bath for 2 h at 95°C. After cooling, the reaction mixture was extracted with CHCl_3_ (3 × 5 ml). The aqueous layer was evaporated to dryness with MeOH until neutral. The dried residue was dissolved in 1 ml anhydrous pyridine and treated with L-cysteine methyl ester hydrochloride (1.5 mg) stirred at 60°C for 1 h. Trimethylsilylimidazole (1.0 ml) was added to the reaction mixture, and kept at 60°C for 30 min.

The reaction mixture was analyzed by GC (Agilent 7890 A), under the following conditions: GC: FID. Column: HP-5 quartz capillary column (30 m × 0.32 mm). Column temperature: 100–230 with the rate of 10°C/min, and the carrier gas was N_2_ (2 ml/min); injector temperature: 250; split ratio: 1/20. The standard monosaccharides were subjected to the same reaction and GC-MS analysis. Under these conditions, the derivatives of D-glucose, L-rhamnose and L-arabinose were detected at 10.942, 7.645, and 8.138 min respectively.

### Preparation and standardisation of inocula

#### Preparation of yeasts inocula

Inoculum of each yeast was prepared from a 48 hours Sabouraud Dextrose Agar culture. Isolated colonies from this culture were diluted in 0.9% NaCl to match the 0.5 Mc Farland standard turbidity, corresponding to about 1.5 × 10^8^ colony forming unit (CFU) per mL. This microbial suspension was diluted to match the 0.09 optical density at 600 nm corresponding to 2.5 × 10^5^ spores.mL^−1^ using a Jenway 6105UV/Vis spectrophotometer (50 Hz/60 Hz) [7].

#### Preparation of dermatophyte inocula

The inoculum of each dermatophyte was prepared from a 15 days old culture on Sabouraud Dextrose Agar (Conda, Madrid, Spain). The culture surfaces were gently scraped and introduced in test tubes containing 10 mL of sterile saline, homogenized for 5 minutes and filtered. The absorbance of the spore suspensions (filtrates) were read at 530 nm and adjusted with sterile distilled water between 0.15 and 0.17 (Jenway 6105UV/Vis spectrophotometer, 50Hz/60Hz) to match 0.6 × 10^6^ -1.4 × 10^6^ CFU.mL^−1^ [8].

### *In vitro* antimicrobial assay

The broth microdilution method [9] was used to determine the minimum inhibitory concentration (MIC) and minimum fungicidal concentration (MFC) of the tested substances using 96 well microplates (Nunclon, Roskilde, Denmark). 96-well plates were prepared by dispensing into each well 100 μL of Sabouraud Dextrose broth for both yeasts and dermatophytes. A volume of 100 μL of each test sample was added into the first wells of the micro-titre plate. Serial two-fold dilutions of these test samples were made. A volume of 100 μL of the above standardized inocula was then added into each well to match approximately 2.5×10^3^ CFU.mL^−1^ for yeast and 10^4^ CFU.mL^−1^ for dermatophytes in a total volume of 200 μL. This gave final concentration range of 0.1 to 0.00078 mg.mL^−1^ for compounds as well as for reference drug (positive control). For every experiment, sterility control (5% v/v aqueous DMSO and broth) and negative control (broth plus inoculum) were included. The content of each well was mixed thoroughly and the micro well plates were covered with the sterile lips and incubated at 37°C for 48 h for yeasts and at 28°C for 5 days for dermatophytes on a plate shaker (Flow Laboratory, Germany) at 300 rpm. After incubation, fungal growth in each well was monitored by observing and comparing the turbidity of the test wells to that of the positive and negative controls. MIC was the lowest concentration of the test substances that prevented visible growth of the microorganisms.

The MFC values were determined by subculturing 50 μL aliquots of the preparations, which did not show any visible growth of the micro-organisms during MIC determinations, into 150 μL of test substance-free SDB. These preparations were further incubated as indicated above. Microbial growth in each well was determined as mentioned above. MFC was the lowest concentration of the test substances that prevented visible growth of the microorganisms in the sub-cultures. All the experiments were performed in triplicates.

## Results

### Structures of isolated compounds

**Figure 1:**
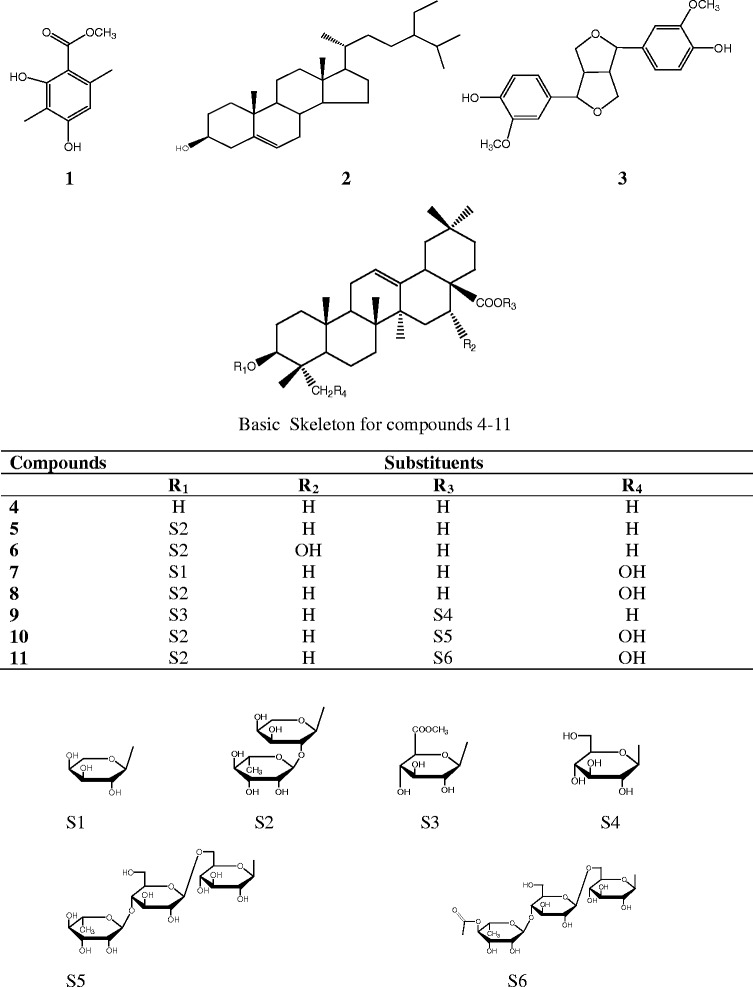
**Chemical structures of compounds isolated from the stem barks of** ***P. fulva.***

Six compounds (**1**–**6**) were isolated from the ethyl acetate fraction (Figure 1); five others (**7**–**11**) from the *n*-butanol fraction, compounds **10** and **11** were also isolated from the residual fraction. The isolated compounds belong to various chemical groups but mainly triterpenoid saponins. The structures of these compounds were established by spectroscopic analysis [IR, ESI-MS, ^1^H and ^13^C NMR spectra in conjunction with 2D experiments (^1^H-^1^H COSY, ROESY, HMBC, and HSQC)] and direct comparison with published data. The compounds were identified as: methyl 2,4-dihydroxy-3,6-dimethylbenzoate (Methyl atrarate) (**1**) [10], *β*-sitosterol (**2**) [11], pinoresinol (**3**) [12], oleanolic acid (**4**) [13], 3-*O*-[*α*-L-rhamnopyranosyl (1–2)-*α*-L-arabinopyranosyl]-oleanolic acid or *β*-hederagenin (**5**) [13], 3-*O*-[*α*-L-rhamnopyranosyl (1–2)-*α*-L-arabinopyranosyl]-echinocystic acid (**6**) [14], 3-*O-α*-L- arabinopyranosyl-hederagenin (**7**) [15], 3-*O*-[*α*-L-rhamnopyranosyl (1–2)-*α*-L-arabinopyranosyl]-hederagenin (**8**) [13], 3-*O*-[methyl-*β*-D-glucurono-pyranosiduronoate]-28-*O-β*-D-glucopyranosyl oleanolate (**9**) [16], 3-*O*-[α-L-rhamnopyranosyl (1–2)-*α*-L-arabinopyranosyl]-28-*O*-[*O*-*α*-L-rhamnopyranosyl (1–4)-*O-β*-D-glucopyranosyl-(1–6)-*β*-D-glucopyranosyl]-hederagenin (**10**) [14]. Compound **11**, 3-*O*-[*α*-L-rhamnopyranosyl (1–2)-*α*-L-arabinopyranosyl]-28-*O*-[*α*-L-4-*O*-acetyl-rhamnopyranosyl (1–4)-*β*-D-glucopyranosyl-(1–6)-*β*-D-glucopyranosyl]-hederagenin appear to be a new compound and fully describe bellow.

### Structure elucidation of compound 11

**Figure 2:**
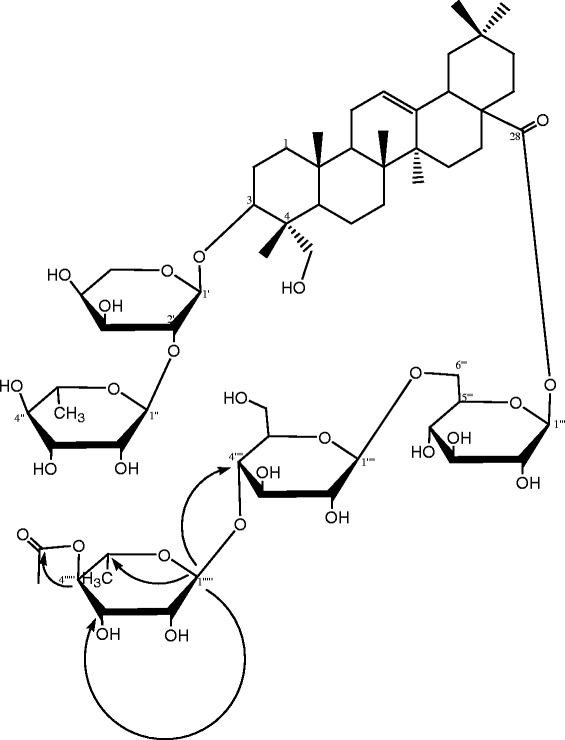
**Structure and key HMBC correlations of compound 11.**

Compound **11** was obtained as a white powder from EtOAc, m.p. 213–215°C, [α]_D_ -38,9 (c 0.1, MeOH) and was positive to Liebermann-Burchard and Molish reaction tests characteristic of triterpenoids and glycosides. The molecular formula was established as C_61_H_98_O_27_ according to HRESI-MS which showed a pseudomolecular ion peak at *m/z* 1285. M + Na]^+^ (Calcd. for C_61_H_98_O_27_Na, 1285.6193). The IR spectrum also showed absorptions at 3428, 1731 and 1642 cm^−1^ accounting for hydroxyl, carbonyl and double bond, repectively. Anomeric proton signals in NMR spectrum at *δ* 5.10 (1H, d, *J* = 6.2 Hz, H-1’), 6.21 (1H, s, H-1”), 6.21 (1H, brd, *J* = 4.2 Hz, H-1”’), 5.00 (1H, d, *J* = 7.8 Hz, H-1””) and 5.83 (1H, s, H-1””’) together with carbon signals at *δ* 104.5 (C-1’), 102.2 (C-1”), 95.7 (C-1”’), 104.9 (C-1””) and 101.7 (C-1””’) in the ^13^C NMR spectrum suggested that compound **11** was a glycoside with five sugar units. By comparing the NMR data with those reported by Maillard *et al*. [17], 23-hydroxy oleanolic acid was also identified as the aglycone. Under the conditions of acid hydrolysis and GC analysis, the derivatives of D-glucose, L-rhamnose and L-arabinose were detected at 10.942, 7.645, and 8.138 min respectively. By comparing with the aglycone in the ^13^C NMR spectrum, C-3 and C-28 were observed distinct down-field or up-field shift respectively indicating that sugar moieties were attached to these two positions. The data of the glycoside moieties are in agreement with those published by Lu *et al*. [18] and were established as 3-*O*-*α*-L-rhamnopyranosyl(1–2)-*α*-L-arabinopyranoside and 28-*O*-*α*-L-rhamnopyronosyl(1–4)-*ß*-D-glucopyranosyl(1–4)-*ß*-D-glucopyranosyde. The presence of an acetyl group was revealed by the presence of two signals in the ^13^C NMR spectrum at *δ* 170.9 and 21.3 which were respectively assigned to a carbonyl group and a methyl, this assumption was further confirmed by a signal in the ^1^H NMR at *δ* 2.01 (3H, s). The relatively deshielded shift of C-4””’ of the L-rhamnopyranosyl of the C-28 unit compare to that of C-4” of the L-rhamnopyranosyl of the C-3 is due to the attachment of the acetyl unit at this position. This information was further supported by the long-range correlation observed between H-4”” and this carbonyl group. The ^1^H and ^13^C NMR data of compound **11** (Table 1) were assigned on the basis of DEPT, ^1^H-^1^H COSY, HSQC and HMBC experiment. Therefore, compound **11** (Figure 2) was characterized as 3-*O*-*α*-L-rhamnopyranosyl(1–2)-*α*-L-arabinopyranosyl-23-hydroxy oleanolic acid-28-*O*-*α*-L-4-*O*-acetyl-rhamnopyranosyl(1–4)-*ß*-D-glucopyranosyl(1–6)-*ß*-D- glucopyranoside or 3-*O*-[*α*-L-rhamnopyranosyl(1–2)-*α*-L-arabinopyranosyl]-28-*O*-[*α*-L-4-*O*-acetyl-rhamnopyranosyl (1–4)-*β*-D-glucopyranosyl-(1–6)-*β*-D-glucopyranosyl]-hederagenin (Figure 2).

### Antifungal properties of compounds isolated from *P. fulva* stem bark

The isolated constituents of *P. fulva* were screened for their antifungal properties against 8 yeasts (7 *Candida* species and *Cyptococcus neoformans*) and **11** dermatophyes (4 *Microsporum*, 6 *Trichophyton* and 1 *Epidermophyton*) (Table 2). Compounds **7** and **8** were relatively active against all the tested yeasts while **2**, **3** and **9** were totally inactive. The antifungal properties of the other compounds were selectively observed on some yeasts. All the tested substances showed a wide range of antidermatophytic activities, inhibiting the growth of almost all the tested dermatophytes (Table 2). Compounds **10** and **11** (MIC generally greater than 50 μg/ml) appeared to be the less actives among all. Compounds **1**–**3** expressed relatively good antidermatophytic activities against *M. ferrugeneum*, *M. audouinii*, *T. violaceum* and *E. flocossum*. Compounds **4** and **5** were the most efficient in inhibiting the dermatophytes growth. Compounds **7** and **8** showed intermediate antidermatophytic activities. Some of these constituents from *P. fulva* extract possess MICs less than the reference drug Griseofulvin on selected microorganisms.

**Table 2:** **MICs/MFCs (μg/ml) of isolated compounds on tested yeasts and dermatophytes**

**Fungi**	**1**	**2**	**3**	**4**	**5**	**6**	**7**	**8**	**9**	**10**	**11**	**Nyst or Gri**
**Yeasts**												
*C. albicans (ATCC 1663)*	−/−	−/−	−/−	−/−	100/-	50/-	100/-	12.5/25	−/−	−/−	−/−	0.04/0.08
*C. Glabrata* (IP 35)	−/−	−/−	−/−	12.5/100	−/−	−/−	12.5/25	12.5/12.5	−/−	100/-	25/100	0.02/0.02
*C. Lucitaniae* (ATCC 200950)	−/−	−/−	−/−	−/−	−/−	−/−	50/50	50/50	−/−	−/−	−/−	0.02/0.08
*C. Parapsilosis (*ATCC 22019)	−/−	−/−	−/−	−/−	−/−	−/−	100/100	50/100	−/−	−/−	−/−	0.01/0.08-
*C. guilliermondii*	−/−	−/−	−/−	12.5/-	−/−	12.5/-	12.5/25	12.5/12.5	−/−	100/-	50/-	0.02/0.08
*C. Krusei* (ATCC 6258)	−/−	−/−	−/−	−/−	−/−	−/−−/−	100/100	25/50	−/−	−/−	−/−	0.04/0.08
*Cryptococcus neoformans* (IP 95026)	100/-	−/−	−/−	−/−	−/−	−/−	12.5/100	6.25/50	−/−	6.25/-	−/−	2.00/2.00
**Dermatophytes**												
*T. ajelloi*	−/−	6.25/12.5	6.25/12.5	6.25/12.5	0.78/1.56	200/200	12.5/12.5	6.25/12.5	200/-	100/-	50/50	0.31/0.31
*T. terrestre* (E 1501)	100/-	3.12/12.5	3.12/6.25	3.12/6.25	3.12/6.25	−/−	12.5/50	12.5/12.5	100/100	−/−	50/50	50/100
*T. equinum*	100/-	100/-	100/-	6.25/100	−/−	−/−	50/100	25/50	100/-	50/100	100/100	0.31/0.31
*T. mentagrophytes* (E 1425)	100/-	100/-	100/-	6.25/100	−/−	−/−	25/25	12.5/25	100/-	50/100	100/100	0.78/0.78
*T. rubrum*	25/50	12.5/100	12.5/50	−/−	12.5/-	6.25/200	6.25/6.25	25/50	12.5/100	50/100	100/100	0.31/0.31
*E. floccosum* (E 1423)	0.78/3.12	0.78/0.78	0.78/0.78	0.78/1.56	0.78/0.78	50/50	25/25	6.25/25	0.78/0.78	50/100	12.5/12.5	0.31/0.31
*M. gypseum*	12.5/12.5	25/100	12.5/50	50/-	25/-	200/-	50/100	12.5/25	25/50	100/100	−/−	1.56/1.56
*M. audouinii*	1.56/3.12	0.78/1.56	0.78/0.78	−/−	0.78/0.78	25/50	12.5/25	6.25/25	0.78/1.56	50/100	12.5/12.5	0.78/0.78
*M. canis* (CBS 113480)	−/−	12.5/200	−/−	25/400	12.5/-	6.25/100	6.25/200	6.25/50	12.5/200	200/400	200/400	0.78/0.78
*M. ferrugeneum* (CBS 471.80)	0.78/1.56	0.78/0.78	0.78/0.78	0.78/1.56	0.78/0.78	25/-	1.56/1.56	0.78/3.12	0.78/0.78	100/100	25/50	0.31/0.31
*T. violaceum* (CBS 201.88)	12.5/50	3.12/100	3.12/50	6.25/-	6.25/-	12.5/200	100/-	1.56/-	6.25/50	25/100	1.56/100	0.31/0.78

Nyst: Nystatin, Gri: Griseofulvin, −: MIC or MFC was greater than 400 μg.

## Discussion

The isolated compounds globally demonstrated more or less interesting antifungal activities. They were phenolics (**1** and **3**), steroids (**2**), triterpene (**4**) and terpenoid saponins (**5**–**11**) secondary metabolites. Most of the antimicrobial substances isolated from Cameroonian medicinal plants belong to these chemical groups [19]. Up to 7 of the 11 isolated compounds from *P. fulva* were terpenoid saponins; such substances with hederagenin or oleanolic acid as aglycone have been found to possess antifungal activities against yeasts and filamentous fungi [20]. Saponins possess the ability to bind with sterols in fungal membrane and cause pore formation and loss of membrane integrity [21] as antifungal of polyene group [22]. Structure-activity relationship of these types of compounds has been demonstrated and their antifungal properties depend on the number and type of sugar residues, but the increase in sugar length does not enhance the activity [21]. The anti-yeasts activities of saponins **7** and **8** (the only active compounds on yeasts) compared to compounds with the same basic skeleton (**4**–**6** and **9–11**) could be ascribed to the presence of a hydroxyl group at position 23 coupled to the presence of a free carboxyl group at position 28. In contrast, all these compounds presented a broad range antidermatophytic activity with compounds **10** and **11** being the less efficient. In fact these last compounds are characterized by the esterification of the carboxyl group (C-28) by 28-*O*-[*O*-*α*-L-rhamnopyranosyl (1–4)-*O-β*-D-glucopyranosyl-(1–6)-*β*-D-glucopyranosyl] (S5) and 28-O-[*α*-L-4-*O*-acetyl-rhamnopyranosyl (1–4)-*β*-D-glucopyranosyl-(1–6)-*β*-D-glucopyranosyl] (S6) respectively. This carboxyl group may play an important role in their antifungal activities.

A number of studies have been carried out on the antifungal activity of phenolic compounds from natural sources [22]. It was the case of pinoresinol (**3**) that has previously been described as fungicidal agent from *Sambucus williamsii* [23] and Methyl atrarate (**1**) that showed a good antifungal activity (MIC 6 μg/ml) on *Candida albicans* [24]. Furthermore antifungal properties of phenolic compounds may be due to iron deprivation or hydrogen binding with vital proteins such as microbial enzymes [25]. According to Hwang *et al.* [23], compound **3** may depolarize or form pores in the fungal bilayer membrane. These two compounds, different in size but with the same number of hydroxyl groups were inactive on yeast and possess almost the same antidermatophytic activities. It is postulated that the site(s) and number of hydroxyl groups of phenolic compounds are closely correlated to their antimicrobial activities [22].

The broad range antidermatophytic activities of the isolated compounds from *P. fulvia* explains the relatively good *in vitro* and *in vivo* antidermatophytic activity of the oil-moistened dichloromethane-methanol (1:1 v/v) crude extract from this plant [6]. They can then serve as markers for the standardization of antidermatophytic phytomedicine from *P. fulva*.

## Conclusion

The tested compounds showed a broad range of antidermatophytic activities while only compounds **7** and **8** inhibited the growth of yeasts. Considering these results and those from our previous studies on the crude extract, these substances may be useful in the standardization of antimicrobial and particularly antidermatophytic phytomedicine from *P. fulva*.
